# Health Care Trainees’ and Professionals’ Perceptions of ChatGPT in Improving Medical Knowledge Training: Rapid Survey Study

**DOI:** 10.2196/49385

**Published:** 2023-10-18

**Authors:** Je-Ming Hu, Feng-Cheng Liu, Chi-Ming Chu, Yu-Tien Chang

**Affiliations:** 1 Division of Colorectal Surgery, Department of Surgery Tri-service General Hospital National Defense Medical Center Taipei Taiwan; 2 Graduate Institute of Medical Sciences National Defense Medical Center Taipei Taiwan; 3 School of Medicine National Defense Medical Center Taipei Taiwan; 4 Division of Rheumatology/Immunology and Allergy, Department of Medicine Tri-Service General Hospital National Defense Medical Center Taipei Taiwan; 5 School of Public Health National Defense Medical Center Taipei Taiwan; 6 Graduate Institute of Life Sciences National Defense Medical Center Taipei Taiwan; 7 Big Data Research Center College of Medicine Fu-Jen Catholic University New Taipei City Taiwan; 8 Department of Public Health Kaohsiung Medical University Kaohsiung Taiwan; 9 Department of Public Health China Medical University Taichung Taiwan

**Keywords:** ChatGPT, large language model, medicine, perception evaluation, internet survey, structural equation modeling, SEM

## Abstract

**Background:**

ChatGPT is a powerful pretrained large language model. It has both demonstrated potential and raised concerns related to knowledge translation and knowledge transfer. To apply and improve knowledge transfer in the real world, it is essential to assess the perceptions and acceptance of the users of ChatGPT-assisted training.

**Objective:**

We aimed to investigate the perceptions of health care trainees and professionals on ChatGPT-assisted training, using biomedical informatics as an example.

**Methods:**

We used purposeful sampling to include all health care undergraduate trainees and graduate professionals (n=195) from January to May 2023 in the School of Public Health at the National Defense Medical Center in Taiwan. Subjects were asked to watch a 2-minute video introducing 5 scenarios about ChatGPT-assisted training in biomedical informatics and then answer a self-designed online (web- and mobile-based) questionnaire according to the Kirkpatrick model. The survey responses were used to develop 4 constructs: “perceived knowledge acquisition,” “perceived training motivation,” “perceived training satisfaction,” and “perceived training effectiveness.” The study used structural equation modeling (SEM) to evaluate and test the structural model and hypotheses.

**Results:**

The online questionnaire response rate was 152 of 195 (78%); 88 of 152 participants (58%) were undergraduate trainees and 90 of 152 participants (59%) were women. The ages ranged from 18 to 53 years (mean 23.3, SD 6.0 years). There was no statistical difference in perceptions of training evaluation between men and women. Most participants were enthusiastic about the ChatGPT-assisted training, while the graduate professionals were more enthusiastic than undergraduate trainees. Nevertheless, some concerns were raised about potential cheating on training assessment. The average scores for knowledge acquisition, training motivation, training satisfaction, and training effectiveness were 3.84 (SD 0.80), 3.76 (SD 0.93), 3.75 (SD 0.87), and 3.72 (SD 0.91), respectively (Likert scale 1-5: strongly disagree to strongly agree). Knowledge acquisition had the highest score and training effectiveness the lowest. In the SEM results, training effectiveness was influenced predominantly by knowledge acquisition and partially met the hypotheses in the research framework. Knowledge acquisition had a direct effect on training effectiveness, training satisfaction, and training motivation, with β coefficients of .80, .87, and .97, respectively (all *P*<.001).

**Conclusions:**

Most health care trainees and professionals perceived ChatGPT-assisted training as an aid in knowledge transfer. However, to improve training effectiveness, it should be combined with empirical experts for proper guidance and dual interaction. In a future study, we recommend using a larger sample size for evaluation of internet-connected large language models in medical knowledge transfer.

## Introduction

ChatGPT is a powerful, cutting-edge large language model (LLM) launched on Nov 30, 2022, developed by OpenAI, an artificial intelligence (AI) research and deployment company. It is a variant of the GPT-3 model, designed explicitly for chatbot and conversational AI applications [1]. It showcased an impressive advancement in the ability to understand and generate human-like text, opening up new possibilities for language models, and has expanded their capability to perform a wide range of tasks. It reflected a significant step forward in language model technology. Its ability to understand and respond naturally to language inputs makes it useful for various tasks, especially in higher education [2].

ChatGPT was trained on diverse online texts, including books, articles, and websites, on topics ranging from news to Wikipedia to fiction, and it received reinforcement learning from human feedback [3,4]. It attempts to incorporate human-like responses during human-to-human conversations. As a result, humans can fine-tune it to become more accurate and capable of understanding details and narrowly pitched questions [5]. It has been used for a variety of natural language processing tasks, such as language translation, text summarization, and answering questions.

ChatGPT quickly gained popularity for its detailed responses and articulate answers across multiple domains of knowledge [6]. It was the first time a powerful chatbot was made freely available, and it is user-friendly for the general public [3]. The release of ChatGPT made enormous impacts on all walks of life. Concerns and debates that arose immediately following the widespread release of ChatGPT appear understandable owing to the well-described phenomenon of innate resistance to any change of the human mind [4]. However, when users provide insufficient information, ChatGPT tends to make assumptions about what the user wants to hear instead of asking clarifying questions. This tendency can lead to unintended consequences and make ChatGPT, as well as other language models like it, a double-edged sword [7].

Despite ChatGPT’s lack of domain-specific training, its knowledge and interpretation ability could pass or nearly pass examinations designed for postgraduate levels of specialization in a wide range of fields, such as medicine, law, and finance [8]. In particular, several articles have discussed its use in improving knowledge interpretation and transfer [9,10]. Regardless, the major concerns of ChatGPT are plagiarism [11] and generating fraudulent but authentic-looking outcomes [12]. Zuccon and Koopman [13] discovered that relying solely on model knowledge, ChatGPT correctly answered 80% of health care questions. The evidence presented in the prompt can heavily influence the correctness of answers, reducing ChatGPT’s accuracy to only 63%. In fact, when evidence is provided, the model often tends to agree with the evidence’s stance, even if the model would produce an answer with the opposite stance if the evidence was not provided in the input [14]. The study highlights the importance of carefully considering prompt knowledge and the potential biases it may introduce. Concerns raised by ChatGPT have yet to be thoroughly investigated. It is still being determined whether or not ChatGPT will alleviate or exacerbate the issues raised by previous chatbots [15]. Therefore, it is critical to understand how to leverage the strengths and weaknesses of ChatGPT to ensure safe use in knowledge interpretation and transfer.

Because of the rapid advancements and massive information flux in medical and health technologies, there is an urgent need for trainees and professionals to acquire new knowledge across multiple domains. It is critical to train them efficiently and effectively in new knowledge and techniques in order to ensure their proficiency in real-world medicine and health care applications. In this study, we aimed to evaluate trainees’ and professionals’ perceptions of ChatGPT-assisted knowledge transfer, using biomedical informatics as an example.

## Methods

### Ethics Approval

This study was approved by the Tri-Service General Hospital Institutional Review Board in Taiwan (202005069). This board was organized and operates in accordance with the International Conference on Harmonization, World Health Organization Good Clinical Practice, and applicable laws and regulations.

### Study Population

In 2023, we used purposeful sampling to include all undergraduate trainees (n=90) and graduate professionals (n=105) in the School of Public Health at the National Defense Medical Center in Taiwan. There were a total of 195 eligible participants. They have all received biomedical informatics training.

### Demonstration Video and Questionnaires

We created a 2-minute demonstration video (Multimedia Appendix 1) introducing ChatGPT and demonstrating five ChatGPT-assisted training scenarios in biomedical informatics: (1) query question, (2) code debugging, (3) query coding examples, (4) creating self-evaluation quizzes, and (5) query training resources. To help participants understand a practical use scenario, we recoded the practical process of using ChatGPT in learning biomedical informatics (Multimedia Appendix 2, Table S1). Data were collected using an online (web- and mobile-based) questionnaire (Multimedia Appendix 3). We emailed the questionnaire to all eligible participants. The questionnaire was designed according to the Kirkpatrick model [16], with four dimensions to understand the thoughts of the students: (1) perceived knowledge acquisition (KA), (2) perceived training motivation (TM), (3) perceived training effectiveness (TE), and (4) perceived training satisfaction (TS). Three experts reviewed and edited the questionnaire, which has 12 questions, including 1 open-ended question. A 5-point Likert scale was adopted for all questionnaire items (from 1=strongly disagree to 5=strongly agree). The content of the questionnaire is shown in Multimedia Appendix 2, Table S2. The questionnaire was edited and collected using Google Forms. After signing an informed consent form, all participants were required to answer the questionnaire immediately after watching the video.

### Thematic Analysis

The participants provided open-ended responses to question 13, “Do you have any other thoughts or suggestions on the application of ChatGPT to biomedical informatics training? Please give us feedback.” Thematic analysis was used to evaluate these open-text data without the use of a specific theoretical lens [17] via the web tool Taguette (Rémi Rampin). We coded and encapsulated the texts into “benefit” and “concern” themes. Finally, the results were displayed as a sunburst plot using the R package *plotly*.

### Hypotheses, Pathway Analysis, and Statistics

**Figure 1:**
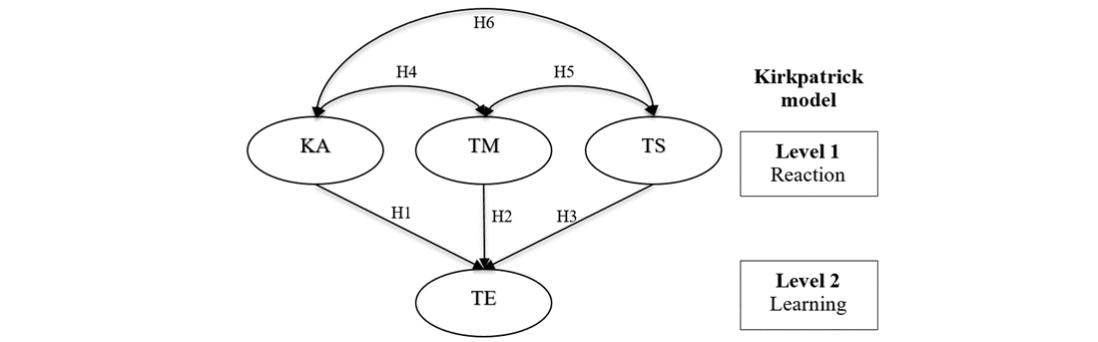
Proposed research framework using Kirkpatrick model for participants’ perception of learning biomedical informatics using ChatGPT. KA: perceived knowledge acquisition; TE: perceived training effectiveness; TM: perceived training motivation; TS: perceived training satisfaction.

The Kirkpatrick model is a well-recognized method of evaluating the results of training effectiveness in 4 levels, including reaction, learning, behavior, and results [16]. This study evaluated the levels of reaction and learning. As depicted, we included a total of 6 hypotheses (H1-H6) to dissect how perceived KA, perceived TM, and perceived TS can together affect the perceived TE. In H1, H2, and H3, we tested whether, with a more positive perception of KA, TM, and TS, the participant would be more likely to have a positive perception of TE. In the other 3 hypotheses (H4-H6), we analyzed the relationship between KA, TM, and TS. In H4, we proposed that KA and TM are positively associated. In H5, we tested whether TS and TM are positively associated. Finally, in H6, we tested whether KA and TS are positively associated (Figure 1).

The association of training evaluation scores between undergraduate trainees and graduate professionals and male and female subjects was analyzed with a 2-tailed Student *t* test. The Kirkpatrick model was analyzed with structural equation modeling (SEM) using R Studio (February 3, 2022, build, version 4.2.0; R Core Team) with the package *lavaan*. The goodness-of-fit indices for the hypothetical model were evaluated as follows: chi-squared test, *P*>.05; relative chi-squared (*χ*^2^/*df*) <2; root mean square error of approximation <0.06; goodness-of-fit index >0.95; and normed fit index >0.90 [18]. SEM is a multivariate statistical analysis technique used to analyze structural relationships. SEM is a method that combines factor analysis and multiple regression analysis. It examines the structural relationship between measured variables and latent constructs. We preferred this method because it estimates multiple and interrelated dependencies in a single analysis [19].

## Results

### Descriptive Statistics

From January to May 2023, we included all eligible participants (N=195). Participants were required to watch the demonstration video before filling out the online questionnaire. The demonstration video enables participants to quickly understand how to use ChatGPT to assist in knowledge transfer and solving problems. There are 5 application scenarios. For example, while previewing the materials, participants can use ChatGPT to ask questions or query difficult codes for a detailed and easy-to-understand explanation. They can use ChatGPT to inquire about specific functional coding, such as how to write a BMI calculation function, or to debug in order to improve their training when writing analysis programming. They can request that ChatGPT create quizzes on specific training topics and provide additional online training resources for self-evaluation and extended training.

Finally, 152 of 195 (75%) participants completed the online questionnaires and were recruited for analysis, with 88 of 152 (58%) being undergraduate trainees and 90 of 152 (59%) being women. The ages ranged from 18 to 53 years (mean 23.3, SD 6.0 years). There was no difference between women and men in perceptions on ChatGPT-assisted learning (Table 1). For both men and women, graduate professionals scored significantly higher on all questions than undergraduate trainees (Table 1). For undergraduate trainees, men and women scored similarly. For graduate professionals, however, men scored significantly higher than women. Male undergraduate trainees and graduate professionals had similar thoughts on question 9: “I believe that by using ChatGPT-assisted training rather than traditional training approaches, I will be more confident in my ability to gain the knowledge and skills being taught” (Table 1). On the other hand, female trainees and professionals had similar thoughts on question 12: “I believe that the information provided by ChatGPT-assisted training meets and exceeds my expectations.”

**Table 1:** Evaluation of perceptions on ChatGPT-assisted training in biomedical informatics. Values for items other than age represent scores on a 5-point Likert scale (from 1=strongly agree to 5=strongly disagree).

Item	Men, mean (SD)		*P* value	Women, mean (SD)		*P* value	All	*P* value^a^
	Undergraduates (n=41)	Graduates (n=21)		Undergraduates (n=47)	Graduates (n=43)			
Age (years)	20.1 (1.27)	28.4 (6.68)	<.001	20.1 (1.42)	27.5 (7.13)	<.001	23.3 (5.98)	.12
Q1: I believe that using ChatGPT to assist with training will allow me to gain more knowledge than traditional training approaches.	3.66 (0.825)	4.48 (0.602)	<.001	3.74 (0.765)	4.09 (0.684)	.03	3.92 (0.785)	.85
Q2: I believe that using ChatGPT to assist training will help me understand the code content better than traditional training approaches.	3.61 (0.862)	4.10 (0.768)	.03	3.60 (0.771)	3.98 (0.707)	.02	3.78 (0.799)	.98
Q3: In comparison to traditional training approaches, I believe that using ChatGPT to assist training will make it easier for me to write the analysis programming I want.	3.61 (0.891)	4.14 (0.910)	.03	3.68 (0.726)	4.00 (0.756)	.04	3.82 (0.825)	.75
Q4: I believe that using ChatGPT-assisted training will increase my desire to do independent training more than traditional training methods.	3.59 (0.974)	4.24 (0.995)	.02	3.43 (0.827)	3.95 (1.05)	.009	3.73 (0.990)	.43
Q5: I believe that using ChatGPT to assist training will increase my motivation to train when compared to traditional training approaches.	3.51 (0.925)	4.14 (0.964)	.02	3.43 (0.773)	4.09 (0.840)	<.001	3.74 (0.912)	.90
Q6: I believe that using ChatGPT to assist with training will help me better understand the complex and difficult parts of training material than traditional training approaches.	3.51 (1.00)	4.14 (0.910)	.02	3.47 (0.776)	3.81 (0.906)	.05	3.67 (0.919)	.54
Q7: I believe that using ChatGPT-assisted training will increase my motivation to practice more when compared to traditional training approaches.	3.63 (0.915)	4.33 (0.856)	.005	3.57 (0.903)	3.95 (0.844)	.04	3.80 (0.914)	.45
Q8: I believe that using ChatGPT-assisted training will benefit my motivation and comprehension of preview training materials more than traditional training approaches.	3.59 (0.865)	4.33 (0.856)	.002	3.49 (0.906)	4.02 (0.801)	.004	3.78 (0.906)	.53
Q9: I believe that by using ChatGPT-assisted training rather than traditional training approaches, I will be more confident in my ability to gain the knowledge and skills being taught.	3.59 (0.999)	4.38 (0.865)	.003	3.57 (0.801)	3.86 (0.774)	.09	3.77 (0.895)	.33
Q10: I believe that by using ChatGPT-assisted training rather than traditional training approaches, I am more confident in my ability to learn programming grammar and data analysis.	3.54 (0.897)	4.38 (0.865)	<.001	3.47 (0.856)	3.91 (0.781)	.01	3.74 (0.897)	.33
Q11: I believe that using ChatGPT-assisted training is more satisfying than traditional training approaches.	3.51 (0.925)	4.10 (0.700)	.02	3.51 (0.882)	3.91 (0.718)	.02	3.70 (0.852)	.95
Q12: I believe that the information provided by ChatGPT-assisted training meets and exceeds my expectations.	3.63 (0.994)	4.05 (0.805)	0.1	3.64 (0.792)	4.07 (0.704)	.008	3.82 (0.849)	.62

^a^This represents a test of the difference in each item between men and women.

When we analyzed the whole group of participants, we found that among the 12 questions the top 2 questions agreed on the most were question 1, “I believe that using ChatGPT to assist with training will allow me to gain more knowledge than traditional training approaches,” and question 3, “In comparison to traditional training approaches, I believe that using ChatGPT to assist training will make it easier for me to write the analysis programming I want”; these questions had scores of 3.92 (SD 0.79) and 3.82 (SD 0.83), respectively (Table 1). On the other hand, the top 2 questions least agreed on were question 6, “I believe that using ChatGPT to assist with training will help me better understand the complex and difficult parts of training material than traditional training approaches,” and question 11, “I believe that using ChatGPT-assisted training is more satisfying than traditional training approaches”; these questions had scores of 3.67 (SD 0.91) and 3.70 (SD 0.85), respectively (Table 1).

In general, trainees and professionals had positive attitudes toward ChatGPT-assisted training in biomedical informatics. The average scores for the 12 questions ranged from 3.67 to 3.92 (SD 0.88; data not shown), higher than the median answer score of 3 points. The average scores for perceived KA, perceived TM, perceived TS, and perceived TE were 3.84 (SD 0.80), 3.76 (SD 0.93), 3.75 (SD 0.87), and 3.72 (SD 0.91), respectively (Table 2). Perceived KA had the highest scores, while perceived TE had the lowest scores. This suggests that ChatGPT-assisted training in biomedical informatics contributed most to KA yet relatively less to TM, TS, and TE.

**Table 2:** Scores for perceptions on the 4 constructs.

Kirkpatrick model/construct			Score, mean (SD)
**Reaction**			
	Perceived training satisfaction	3.75 (0.87)	
	Perceived training motivation	3.76 (0.93)	
**Learning**			
	Perceived knowledge acquisition	3.84 (0.80)	
	Perceived training effectiveness	3.72 (0.91)	

### Open-Ended Responses

**Figure 2:**
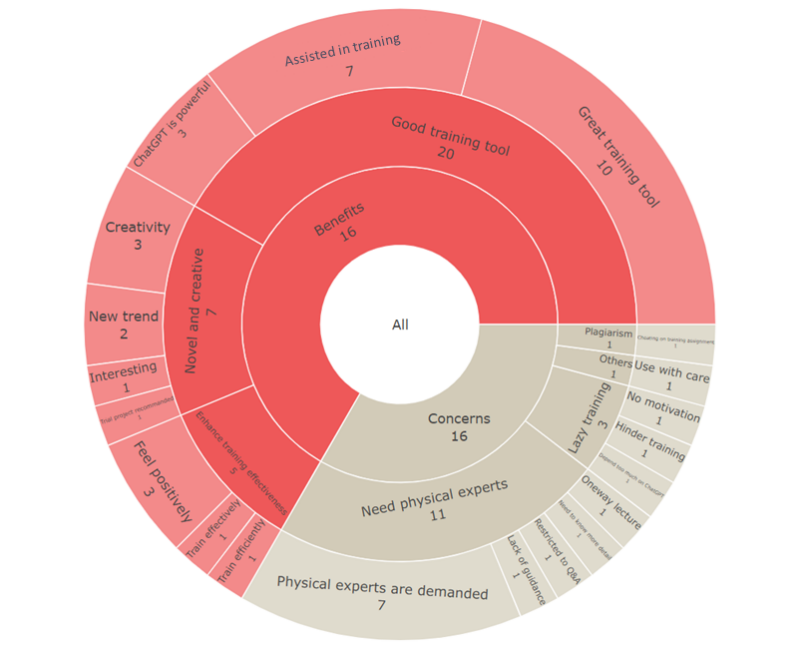
The perceived benefits of and concerns regarding ChatGPT-assisted in training biomedical informatics. The categories presented were defined by thematic analysis of open-ended answers. The numbers in the plot represent the number of participants who elicited each theme.

We included an open-ended question (question 13), “Do you have any other thoughts or suggestions on the application of ChatGPT to biomedical informatics training? Please give us feedback,” in addition to the 12 questions in our online questionnaire. We conducted a thematic analysis on the responses of the 25 participants who answered this question (Multimedia Appendix 2, Table S3). They identified 32 of 48 (67%) potential benefits of ChatGPT-assisted training in biomedical informatics (Figure 2). They believed ChatGPT is novel and creative and a good training tool. For example, one subject said, “Running the trial training program is highly recommended. I eagerly anticipate its implementation. However, it is unfortunate that my graduation is imminent. Nevertheless, this remarkable robot possesses the ability to engage in conversations and seamlessly continue previous discussions, making it feel like interacting with a real interlocutor rather than a mere automaton. Furthermore, ChatGPT can also compose captivating poems and articles upon request. Its impressive capabilities and potential for learning make it an immensely powerful entity deserving of our attention and study.”

On the other hand, some participants identified 16 of 48 (33%) potential concerns about ChatGPT-assisted training in biomedical informatics. They believed that physical empirical experts were still required to provide guidance, and thought that dual-track interaction would result in effective knowledge transfer. For example, a subject stated, “Although self-study can be used to gain knowledge, I believe that we still need an expert to lead and guide us so that we can learn the knowledge effectively. ChatGPT can be useful as an auxiliary tool, but it is limited to the question-and-answer mode of communication. To be able to explore a subject in depth, a physical expert’s guidance is still required...” Furthermore, this subject was concerned that relying too heavily on ChatGPT would impair self-reflective ability and lead to cheating on training evaluations and that ChatGPT should be used with caution to avoid fraudulent prompts. Quantitative and qualitative results showed that trainees and professionals were generally positive and excited about using ChatGPT as a training tool. Most of them were excited about the potential of ChatGPT to enhance knowledge transfer.

### Hypothesis Test

SEM was used to investigate the association between the postulated latent constructs of perceptions on ChatGPT-assisted training based on the Kirkpatrick model. We optimized the path model by adding significant paths and deleting insignificant paths. The goodness-of-fit indices are presented in Multimedia Appendix 2, Table S4. The SEM met all the assumption criteria. The total explainable variation of 4 dimensions by the model was 0.84. The latent construct to which the equations should belong was analyzed and defined by SEM. Part of the questions were categorized differently than in the original design.

**Figure 3:**
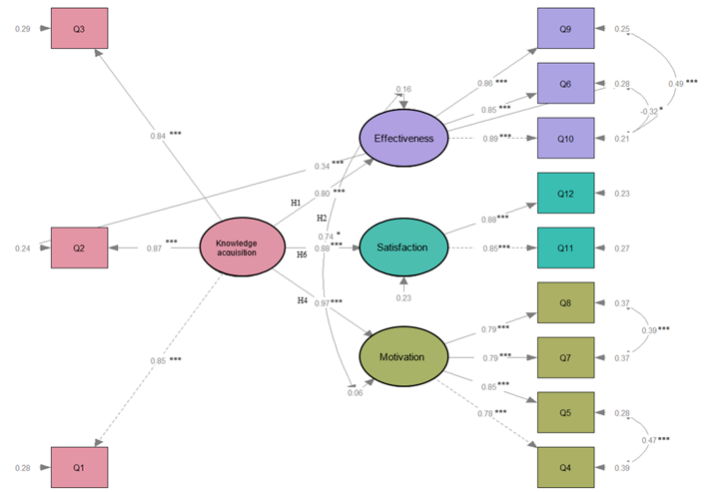
Results of a path analysis of perceptions on ChatGPT-assisted training in biomedical informatics. The coefficients are standardized. Values on the lines represent β values. *P<.05; **P<.01; ***P<.001.

The results of the research model are shown in Table 3 and Figure 3. Participants with more positive perceptions of KA were more likely to have more positive perceptions of TE (H1 β=.80, *P*<.001). However, perceived TM (H2) and perceived TS (H3) did not significantly influence perceived TE. Instead of the expected directed association, perceived TM and perceived TE had a nondirected association (H2 β=.74, *P*=.01). Perceived KA influenced both perceived TM (H4 β=.97, *P*<.001) and perceived TS (H6 β=.88, *P*<.001). Unexpectedly, there was no significant association between perceived TS and perceived TM (H5). These results indicate that the perceived TE of the ChatGPT-assisted training in biomedical informatics was influenced predominantly by perceived KA and partially met the hypotheses in the research framework (Figure 1).

**Table 3:** The results of research model on ChatGPT-assisted training.

Model	Hypothesis	Standardized coefficient	*P* value	Results
H1	Participants with more positive perceptions of knowledge acquisition are more likely to have more positive perceptions of training effectiveness.	.80	<.001	Supported
H2	Participants with more positive perceptions of training motivation are more likely to have more positive perceptions of training effectiveness.	.74	.01	Not supported but nondirected association was discovered
H3	Participants with more positive perceptions of training satisfaction are more likely to have more positive perceptions of training effectiveness.	N/A^a,b^	N/A^b^	Not supported
H4	Perceptions of knowledge acquisition are positively associated with perceptions of training motivation.	.97	<.001	Supported
H5	Perceptions of training motivation are positively associated with perceptions of training satisfaction.	N/A^b^	N/A^b^	Not supported
H6	Perceptions of knowledge acquisition are positively associated with perceptions of training satisfaction.	.88	<.001	Supported

^a^N/A: not applicable.

^b^Due to the lack of statistical significance, these connections were excluded from the model. Consequently, no statistical data are available.

## Discussion

### Principal Findings

This study is an initiative to evaluate the perceptions of health care trainees and professionals on ChatGPT-assisted knowledge transfer concerning biomedical informatics using a hypothesis-driven online questionnaire. We also carried out further thematic, statistical, and pathway analyses. First, the ChatGPT-assisted training received the highest score in perceived knowledge acquisition but the lowest in perceived TE using the Kirkpatrick model. Secondly, graduate professionals expressed more positive attitudes toward the ChatGPT-assisted course than undergraduate trainees. Finally, there was no statistically significant difference in response scores between men and women. The participants agreed that ChatGPT could help them gain more knowledge and ease their training, but they thought that physical empirical experts were still required to provide guidance and that a dual-track interaction would result in more effective knowledge transfer.

Although ChatGPT is a powerful tool for dealing with knowledge-based questions, it has limitations. First, it cannot generate content based on events after its most recent internet scan (in 2021). Therefore, some of the references it generates require updates. Secondly, it occasionally generates errors in math problems or word choice [9]. Since it is a brand-new technology, the model still requires further training. Specifically, ChatGPT may provide false answers [20]. From time to time, ChatGPT provides plausible-sounding but incorrect or nonsensical answers, such as citing a scientific study that does not exist [21]. Furthermore, AI can generate completely fabricated scientific articles that appear sophisticated and flawless [12]. Constraints and bias in the training data can have an adverse effect on the output of the model [21,22]. Third, instead of engaging in genuine conversation, it is often seen that ChatGPT passively follows instructions. For instance, when the information the user provides needs to be more comprehensive, ChatGPT frequently makes assumptions about what the user wants to hear instead of asking clarifying questions [7]. As to medical utility, ChatGPT can provide highly consistent but comparable quality medical information with 60% accuracy [23]. ChatGPT-3.5 had high accuracy in responding to common lung cancer questions; however, neither ChatGPT nor Google Bard, Bing, or the Google search engine answered all questions correctly 100% of the time [24].

ChatGPT was not originally designed for health care applications, and one of the major concerns was response reliability. Trust is crucial for users to adopt ChatGPT. Excessive trust in AI-driven chatbots like ChatGPT carries risks. Relying on ChatGPT for health advice could lead to misinformation and health risks. Efforts should focus on improving ChatGPT’s ability to distinguish between appropriate and inappropriate queries, redirecting the latter to human experts [25]. However, language models are few-shot learners. Few-shot learners are machine learning models that can learn from a limited amount of labeled data and make accurate predictions based on previously unseen examples [26]. The abovementioned limitations of ChatGPT can be improved by few-shot learning. Besides, other upcoming LLMs and generative AIs, such as GPT-4 and Google Bard, have developed their AI models to access the internet. These features ensure that AIs provide up-to-date information for each question asked and that training data is added via the internet to improve response accuracy.

However, relying solely on ChatGPT-assisted training is insufficient for increasing training effectiveness. The quality of ChatGPT responses is heavily dependent on the user’s querying skill. Although ChatGPT can quickly provide the desired information, it lacks the ability to organize and integrate training materials that are best suited to individual aptitudes. Thus, the combination of ChatGPT and empirical experts’ guidance would enhance knowledge transfer. Zachary and Pardos [27] compared the efficacy of ChatGPT hints with hints authored by human tutors among 77 participants across 2 algebra topic areas: elementary algebra and intermediate algebra. Although both human and ChatGPT hints produced positive knowledge transfer, human-created hints produced significantly higher knowledge transfer than ChatGPT hints in both topic areas.

We present a pilot study to assess users’ perceptions of ChatGPT-assisted knowledge transfer in biomedical informatics. We started this study with health care students because they are health care trainees and professionals in this field. Although the sample size is insufficient to generalize the findings to other fields of expertise or users, it did provide a paradigm of ChatGPT-assisted training in new knowledge and techniques. We can foresee the flourishing development of AI chatbots being applied in medicine or health care [28-36]. ChatGPT represents a paradigm shift in the field of virtual assistants. Its successors, including ChatGPT-4, hold great potential as valuable tools for both patients and health care providers. The use of ChatGPT emphasizes the pressing need for regulators and health care professionals to actively participate in the development of minimum quality standards and to enhance patient awareness regarding the current limitations of emerging AI assistants [37]. Thus, LLMs still have a considerable journey ahead before they can be effectively used in the medical or health care domains. The demand for regulations concerning patient privacy and legal responsibilities becomes apparent. Additionally, AI-generated responses must undergo rigorous quality control and review processes. Moreover, the establishment of clinical guidelines is essential to ensure the appropriate use and implementation of these assistants in medical practice [38].

### Limitations

The main limitation of this study is that we focused on the participants’ perceptions of ChatGPT-assisted TE rather than their actual training performance. Furthermore, because the sample size was small, the generalizability of results may be limited. The missing response rate of 22% (43/195) could have resulted in sampling bias. Importance-performance analysis and the Chuchiming index [39] are recommended to determine the demand for and importance of LLM-assisted knowledge transfer for users. Future research should consider actual training performance before and after the intervention, as well as include a larger sample size.

### Conclusions

Most trainees and professionals were enthusiastic about the ChatGPT-assisted knowledge transfer. They agreed that ChatGPT could help them gain more knowledge and ease their training. However, they thought that physical empirical experts were still required to provide guidance and that a dual-track interaction would result in more effective knowledge transfer. In a future study, a larger sample size and an evaluation of an internet-connected LLM for medical knowledge transfer are recommended.

## Appendix

Multimedia Appendix 1 Demonstration video of ChatGPT application.

Multimedia Appendix 2 Tables S1 to S4, including samples and additional statistics.

Multimedia Appendix 3 Web- and mobile-based questionnaire.

## Abbreviations

AI: artificial intelligence
KA: knowledge acquisition
LLM: large language model
TE: training effectiveness
TM: training motivation
TS: training satisfaction

## Data Availability

The data that support the findings of this study are available from the corresponding author upon reasonable request.

## References

[1] Prusinkiewicz K How can we improve language models using reinforcement learning? ChatGPT case study. Deepsense.

[2] Atlas S ChatGPT for higher education and professional development: a guide to conversational AI. DigitalCommons@URI.

[3] Rudolph J, Tan S, Tan S The brilliance and weirdness of ChatGPT. New York Times.

[4] Sallam M The utility of ChatGPT as an example of large language models in healthcare education, research and practice: systematic review on the future perspectives and potential limitations. medrXiv.

[5] Schwartz E OpenAI promises customizable ChatGPT after bias complaints. Voicebot.

[6] Rudolph J, Tan S, Tan S (2023). ChatGPT: Bullshit spewer or the end of traditional assessments in higher education?. J Appl Learn Teach.

[7] Shen Y, Heacock L, Elias J, Hentel KD, Reig B, Shih G, Moy L (2023). ChatGPT and other large language models are double-edged swords. Radiology.

[8] Varanasi L OpenAI just announced GPT-4, which can pass everything from a bar exam to AP Biology with flying colors. Here's a list of difficult exams both AI models have passed. Business Insider.

[9] Lieberman M What Is ChatGPT and how is it used in education?. EducationWeek.

[10] Ofgang E What is ChatGPT and How can you teach with it? Tips and tricks. Tech & Learning.

[11] Bagshaw J, Knight C, Kernohan D ChatGPT, assessment and cheating – have we tried trusting students?. Wonkhe.

[12] Májovský Martin, Černý Martin, Kasal M, Komarc M, Netuka D (2023). Artificial intelligence can generate fraudulent but authentic-looking scientific medical articles: Pandora's box has been opened. J Med Internet Res.

[13] Zuccon G, Koopman B Dr ChatGPT, tell me what I want to hear: How prompt knowledge impacts health answer correctness. arXiv.

[14] White J, Fu Q, Hays S, Sandborn M, Olea C, Gilbert H A prompt pattern catalog to enhance prompt engineering with ChatGPT. arXiv.

[15] Tlili A, Shehata B, Adarkwah MA, Bozkurt A, Hickey DT, Huang R, Agyemang B (2023). What if the devil is my guardian angel: ChatGPT as a case study of using chatbots in education. Smart Learn Environ.

[16] Smidt A, Balandin S, Sigafoos J, Reed VA (2009). The Kirkpatrick model: A useful tool for evaluating training outcomes. J Intellect Dev Disabil.

[17] Tran V, Riveros C, Ravaud P (2019). Patients' views of wearable devices and AI in healthcare: findings from the ComPaRe e-cohort. NPJ Digit Med.

[18] Hooper D, Coughlan J, Mullen M (2008). Structural equation modelling: Guidelines for determining model fit. Electron J Bus Res Methods.

[19] Structural equation modeling. Statistics Solutions.

[20] What is ChatGPT? Top capabilities and limitations you must know. Emeritus.

[21] Thorp HH (2023). ChatGPT is fun, but not an author. Science.

[22] Baidoo-Anu D, Owusu Ansah L (2023). Education in the era of generative artificial intelligence (AI): Understanding the potential benefits of chatgpt in promoting teaching and learning. SSRN Journal.

[23] Walker HL, Ghani S, Kuemmerli C, Nebiker CA, Müller Beat Peter, Raptis DA, Staubli SM (2023). Reliability of medical information provided by ChatGPT: assessment against clinical guidelines and patient information quality instrument. J Med Internet Res.

[24] Rahsepar AA, Tavakoli N, Kim GHJ, Hassani C, Abtin F, Bedayat A (2023). How AI responds to common lung cancer questions: ChatGPT vs Google Bard. Radiology.

[25] Choudhury A, Shamszare H (2023). Investigating the impact of user trust on the adoption and use of ChatGPT: Survey analysis. J Med Internet Res.

[26] Brown T, Mann B, Ryder N, Subbiah M, Kaplan J, Dhariwal P (2020). Language models are few-shot learners. Advances in Neural Information Processing Systems.

[27] Zachary A, Pardos Sb Learning gain differences between ChatGPT and human tutor generated algebra hints. arXiv.

[28] Calleja-López J R Tadeo, Rivera-Rosas CN, Ruibal-Tavares E (2023). Impact of ChatGPT and artificial intelligence in the contemporary medical landscape. Arch Med Res.

[29] Janamala V, Ram IS, Daram SB (2023). Realization of green 5G cellular network role in medical applications: use of ChatGPT-AI. Ann Biomed Eng.

[30] Jin Q, Leaman R, Lu Z (2023). Retrieve, summarize, and verify: How will ChatGPT affect information seeking from the medical literature?. J Am Soc Nephrol.

[31] Kleebayoon A, Wiwanitkit V (2023). ChatGPT in medical practice, education and research: malpractice and plagiarism. Clin Med (Lond).

[32] Koga S (2023). The potential of ChatGPT in medical education: Focusing on USMLE preparation. Ann Biomed Eng.

[33] Li S (2023). Exploring the clinical capabilities and limitations of ChatGPT: a cautionary tale for medical applications. Int J Surg.

[34] Shay D, Kumar B, Bellamy D, Palepu A, Dershwitz M, Walz JM, Schaefer MS, Beam A (2023). Assessment of ChatGPT success with specialty medical knowledge using anaesthesiology board examination practice questions. Br J Anaesth.

[35] Tsang R (2023). Practical applications of ChatGPT in undergraduate medical education. J Med Educ Curric Dev.

[36] Zhou J, Jia Y, Qiu Y, Lin L (2023). The potential of applying ChatGPT to extract keywords of medical literature in plastic surgery. Aesthet Surg J.

[37] Hopkins AM, Logan JM, Kichenadasse G, Sorich MJ (2023). Artificial intelligence chatbots will revolutionize how cancer patients access information: ChatGPT represents a paradigm-shift. JNCI Cancer Spectr.

[38] Mesko B (2023). The ChatGPT (generative artificial intelligence) revolution has made artificial intelligence approachable for medical professionals. J Med Internet Res.

[39] Cheng C, Huang Y, Sun C, An C, Chang Y, Chu C, Chang C (2022). Gender-specific determinants of eHealth literacy: Results from an adolescent internet behavior survey in Taiwan. Int J Environ Res Public Health.

