# Supplementary material for: Health Care Trainees’ and Professionals’ Perceptions of ChatGPT in Improving Medical Knowledge Training: Rapid Survey Study
Source: J Med Internet Res. 2023 Oct 18;25:e49385. doi: 10.2196/49385 (PMC10620632; doi:10.2196/49385)
Supplement: Multimedia Appendix 2 [file jmir_v25i1e49385_app2.pdf]

Table S 1 The video contained demonstration scenarios of ChatGPT-assisted training in biomedical informatics.

Scenario	Video Content
Query question	Please explain what is boolean value?<br><br>A boolean value is a data type that represents the truth values of logic and can only have one of two possible states: true or false. The term "boolean" is named after the mathematician and logician George Boole, who developed Boolean algebra, a branch of mathematics that deals with logical operations.<br><br>Boolean values are fundamental in computer programming and are used extensively in conditional statements, decision-making, and logical operations. They serve as the building blocks for logical expressions and allow programmers to control the flow of execution in their programs.<br><br>In programming languages, boolean values are often represented by the keywords "true" and "false". These values are used to express the logical outcome of comparisons, conditions, and operations. For example, in an if statement, the condition is evaluated, and if it is true, the corresponding block of code is executed. If the condition is false, the code block is skipped.<br><br>Boolean values can be combined using logical operators such as "and", "or", and "not" to create more complex conditions. The "and" operator returns true if both operands are true, the "or" operator returns true if at least one operand is true, and the "not" operator negates the boolean value, returning its opposite.
Coding debugging	Please tell me what's wrong with the R code "<br><br>sub(){<br>c=a+b<br>return(c)<br>}<br><br><br>"

There are a few issues with the provided R code:

1. Function definition: The code starts with `sub()`, which suggests that it is intended to define a function. However, the function name is missing, and parentheses are empty. A valid function definition in R should have a name and may include parameters within the parentheses.
2. Variable assignment: The statement `c = a + b` tries to assign the value of the sum of variables `a` and `b` to the variable `c`. However, `c` is a reserved function in R used for concatenation. It's best to avoid using reserved function names as variable names to prevent conflicts.
3. Return statement: The `return()` function is used to specify the value to be returned from a function. However, in this code, it is unnecessary to use `return()` since the last expression in a function is automatically returned.

To correct these issues, you can modify the code as follows:

R Copy code

```r
myFunction <- function(a, b) {
  c <- a + b
  c
}
```

In the corrected code:

- The function is defined with the name `myFunction` and takes two parameters `a` and `b`.
- The sum of `a` and `b` is assigned to the variable `c`.
- The value of `c` is automatically returned without using `return()`.

query coding examples

Please give me a BMI calculation function using R code

Certainly! Here's an example of an R function to calculate the Body Mass Index (BMI) based on weight and height:

R Copy code

```r
calculateBMI <- function(weight, height) {
  bmi <- weight / (height^2)
  bmi
}
```

<table>
<tr>
<td></td>
<td>

In the above code:

- The `calculateBMI` function is defined with two parameters: `weight` and `height`.
- The BMI is calculated by dividing the weight (in kilograms) by the square of the height (in meters).
- The calculated BMI value is stored in the variable `bmi`.
- The value of `bmi` is returned as the output of the function.

You can use this function by passing the weight and height values as arguments. For example:

R Copy code

```
weight <- 75  # in kilograms
height <- 1.8  # in meters

result <- calculateBMI(weight, height)
print(result)
```

In the above code, the weight is set to 75 kilograms and the height to 1.8 meters. The `calculateBMI` function is called with these values, and the result is stored in the `result` variable. Finally, the result is printed to the console.

</td>
</tr>
<tr>
<td>Creating self-evaluation quizzes</td>
<td>

語恬 Please give me five quizzes ablout the loop in R language

</td>
</tr>
</table>

Sure! Here are five quiz questions about loops in the R programming language:

1. Question: Which loop construct in R is used when the number of iterations is known in advance?
   A) for loop
   B) while loop
   C) repeat loop
   D) if-else statement
2. Question: What is the purpose of the `break` statement in a loop?
   A) It terminates the loop and transfers control to the next iteration.
   B) It skips the current iteration and proceeds to the next iteration.
   C) It is used to define the exit condition for the loop.
   D) It reverses the loop's control flow.
3. Question: What is the purpose of the `next` statement in a loop?
   A) It terminates the loop and transfers control to the next iteration.
   B) It skips the current iteration and proceeds to the next iteration.
   C) It is used to define the exit condition for the loop.
   D) It reverses the loop's control flow.

4. Question: Which loop construct in R is suitable for iterating over elements of a vector or a list?
   A) for loop
   B) while loop
   C) repeat loop
   D) if-else statement
5. Question: What is the purpose of the `while` loop in R?
   A) It allows iterating a specific number of times.
   B) It repeatedly executes a block of code until a condition becomes false.
   C) It is used to check a single condition and perform different actions based on its outcome.
   D) It is a control statement used in error handling.

Answers:

1. A) for loop
2. A) It terminates the loop and transfers control to the next iteration.
3. B) It skips the current iteration and proceeds to the next iteration.
4. A) for loop
5. B) It repeatedly executes a block of code until a condition becomes false.

Query training resources	Please suggest online learning tutorials for R learners<br><br>Certainly! Here are some online learning tutorials and resources that can be helpful for R learners:<br><br>1. R for Data Science (r4ds.had.co.nz): This online book by Hadley Wickham and Garrett Grolemund provides a comprehensive introduction to data science with R. It covers essential concepts, tools, and techniques for working with data using R.<br>2. DataCamp (www.datacamp.com): DataCamp offers interactive R courses for all skill levels. Their courses cover a wide range of topics, including data manipulation, data visualization, statistical analysis, machine learning, and more.<br>3. R Programming (www.coursera.org/learn/r-programming): This Coursera course, part of the Data Science Specialization from Johns Hopkins University, provides a solid foundation in R programming. It covers the basics of R, data structures, control structures, functions, and data cleaning.

Table S 2 The internet questionnaire

No.	Construct	Question
Q1	Perceived knowledge acquisition	I believe that using ChatGPT to assist with training will allow me to gain more knowledge than traditional training approaches.
Q2		I believe that using ChatGPT to assist training will help me understand the code content better than traditional training approaches.
Q3		In comparison to traditional training approaches, I believe that using ChatGPT to assist training will make it easier for me to write the analysis programming I want.
Q4	Perceived training motivation	I believe that using ChatGPT-assisted training will increase my desire to do independent training more than traditional training methods.
Q5		I believe that using ChatGPT to assist training will increase my motivation to train when compared to traditional training approaches.
Q7		I believe that using ChatGPT-assisted training will increase my motivation to practice more when compared to traditional training approaches.
Q8		I believe that using ChatGPT-assisted training will benefit my motivation and comprehension of preview training materials more than traditional training approaches.
Q6	Perceived training effectiveness	I believe that using ChatGPT to assist with training will help me better understand the complex and difficult parts of training material than traditional training approaches.
Q9		I believe that by using ChatGPT-assisted training rather than traditional training approaches, I will be more confident in my ability to gain the knowledge and skills being taught.
Q10		I believe that by using ChatGPT-assisted training rather than traditional training approaches, I am more confident in my ability to learn programming grammar and data analysis.
Q11	Perceived training satisfaction	I believe that using ChatGPT-assisted training is more satisfying than traditional training approaches.
Q12		I believe that the information provided by ChatGPT-assisted training meets and exceeds my expectations.

Q13	Open-ended question	Do you have any other thoughts or suggestions on the application of ChatGPT to biomedical informatics training? Please give us feedback.

Table S 3 The responses on the open-ended question

1	It is recommended to have a basic introductory course understanding of programming language, and the self-learning effect with ChatGPT will be better.
2	ChatGPT seems to be a one-way training? If I have any question, then ask physical experts?
3	I want to know the scope of ChatGPT-assisted training and application
4	I think ChatGPT can benefit programming languages training and achieve the desired goals.
5	I think this is an application that conforms to today's information trends, and it is of great help in gaining knowledge.
6	Although self-study can be used to gain knowledge, I believe that we still need an expert to lead and guide us so that we can learn the knowledge effectively. ChatGPT can be useful as an auxiliary tool, but it is limited to the question-and-answer mode of communication. To be able to explore a subject in depth, a physical expert's guidance is still required. Thanks for your hard work!
7	I'm a slacker learner. Even with the help of good tools like ChatGPT, I may still be lacking in motivation; however, if there are evaluation assignments or exams, I will be more willing to use ChatGPT.
8	I think it is very novel, but I am afraid that someone will use ChatGPT to solve evaluation assignments.
9	I believe the development of such a useful tool facilitates knowledge transfer!
10	I believe that traditional training methods and ChatGPT-assisted training can run concurrently.
11	ChatGPT-assisted training, in my opinion, still has limitations. There is no syllabus or guideline for trainees, and no reference for evaluating training effectiveness. People with good memories continue to have a significant advantage when it comes to gaining knowledge.
12	Running the trial training program is highly recommended. I eagerly anticipate its implementation. However, it is unfortunate that my graduation is imminent. Nevertheless, this remarkable robot possesses the ability to engage in conversations and seamlessly continue previous discussions, making it feel like interacting with a

	real interlocutor rather than a mere automaton. Furthermore, ChatGPT can also compose captivating poems and articles upon request. Its impressive capabilities and potential for learning make it an immensely powerful entity deserving of our attention and study.
13	ChatGPT can give this program greater attention
14	After all, ChatGPT's database is 530TB in size. It's fine to ask him if you don't understand the training materials. It is significantly faster than searching for answers on one's own. You can also revise the generated response using your imagination and creativity..
15	It's a great idea
16	Both ChatGPT and traditional training methods are acceptable.
17	ChatGPT-assisted training sounds more helpful.
18	It feels good, but some training materials may still need to be explained by physical experts in a more colloquial and simple manner. Furthermore, physical experts may have their own experience to speak about things that this app cannot provide.
19	Although learning a programming language can be done with the help of artificial intelligence, I would prefer to be trained by a physical expert if it is truly necessary. If I use artificial intelligence, I am concerned about dependence and a lack of self-reflective ability.
20	AI answers should be interpreted and used with caution.
21	Contacting AI as soon as possible can help you better understand future technological trends.
22	The training method for incorporating AI is very appealing.
23	ChatGPT should be a good training tool.
24	To maximize the benefits, it is still necessary to collaborate with physical experts.
25	The application of ChatGPT in programming languages is often done on Azure AI, where it is developed and operated. It leverages cloud computing capabilities for efficient data processing and offers a wide range of available packages and libraries. This makes it an excellent training tool for exploring and mastering programming languages.

Table S 4 Goodness-of-fit indices for the SEM model

**Fit index**	**Value**	**Recommended value**
X^2/df [a]	85.868/44=1.95	1<NC<3
P [a]	<.001	>.50
SRMR	.029	<.08
RMSEA	.079	<.05
GFI	.918	>.90
NFI	.952	>.90
NNFI	.964	>.90
CFI	.976	>.95

df: Degree of freedom, SRMR=Standardized root mean square residual, RMSEA: Root mean square error of approximation, GFI=Goodness-of-fit index, NFI: Normed fit index, NNFI=Non-normed fit index, CFI=Comparative fit index. [a] At least one out of these two indexes meets the recommended values are required.
