# Supplementary material for: Health Care Trainees’ and Professionals’ Perceptions of ChatGPT in Improving Medical Knowledge Training: Rapid Survey Study
Source: J Med Internet Res. 2023 Oct 18;25:e49385. doi: 10.2196/49385 (PMC10620632; doi:10.2196/49385)
Supplement: Multimedia Appendix 3 [file jmir_v25i1e49385_app3.pdf]

chat-GPT輔助學習評估

感謝您協助填寫本問卷，以助於我們了解學生對於chat-GPT用於輔助新知識學習的認知，本問卷設計以生物資訊知識學為範例，您可以自行選擇是否填寫此問卷，本問卷之資料僅做學術研究使用不另做其他用途，問卷資料將由計畫主持人以電子檔形式密碼保存於雲端，以確保資料保密不外流。請觀看2分鐘影片後填寫問卷，問卷填寫僅需花費約2分鐘，感謝您參與本研究。

如有任何問題歡迎聯絡計畫主持人:
國防醫學院公衛系 張語恬 老師
02-87923100#18454
greengarden720925@gmail.com

* 表示必填問題

1。 電子郵件 *

____

請觀賞約2分鐘的影片後填寫問卷，感謝您

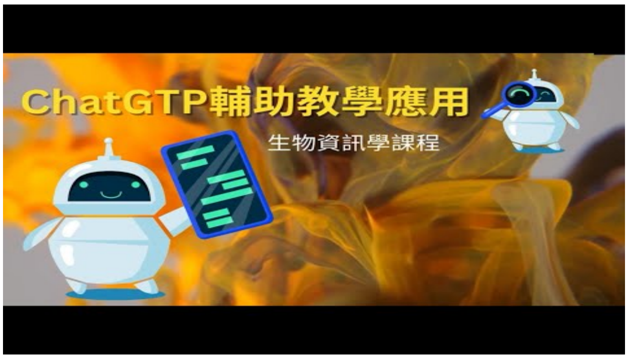

http://youtube.com/watch?v=6BD0NerrgI8

基本資料

2。 請問您的身分是 *

單選。

- ( ) 大學部學生
- ( ) 碩士生
- ( ) 博士生

3。 性別 *

單選。

- 生理男
- 生理女

4。 年齡（年） *

____

一、知識獲得

5。 我認為使用chat-GPT輔助教學相較於傳統老師授課，能使我獲得更多課程相關的知識 *

單選。

非常不同意

1 ◯

2 ◯

3 ◯

4 ◯

5 ◯

非常同意

6。 我認為使用chat-GPT輔助教學相較於傳統老師授課，能使我更容易理解程式碼內容 *

單選。

非常不同意
1
2
3
4
5
非常同意

7. 我認為使用chat-GPT輔助教學相較於傳統老師授課，能使我更容易撰寫出我想要的分析語法 *

單選。

	非常不同意
1	⬭
2	⬭
3	⬭
4	⬭
5	⬭
	非常同意

二、學習動機與成效

8。 我認為使用chat-GPT輔助教學相較於傳統老師授課，能使我更願意自主學習程式語言 *

單選。

非常不同意

1 ⬭

2 ⬭

3 ⬭

4 ⬭

5 ⬭

非常同意

9。 我認為使用chat-GPT輔助教學相較於傳統老師授課，能更提升我學習程式語言的動機 *

單選。

	非常不同意
1	⬭
2	⬭
3	⬭
4	⬭
5	⬭
	非常同意

10。 我認為使用chat-GPT輔助教學相較於傳統老師授課，能使我更理解程式語法中複雜難懂的部分 *

單選。

1 ◯

2 ◯

3 ◯

4 ◯

5 ◯

11。 我認為使用chat-GPT輔助教學相較於傳統老師授課，能更增加我在非課堂時練習語法撰寫的動力 *

單選。

1

2

3

4

5

12。 我認為使用chat-GPT輔助教學相較於傳統老師授課，能更有助於我課前預習的動機與理解 *

單選。

非常不同意
1 ◯
2 ◯
3 ◯
4 ◯
5 ◯
非常同意

13。 我認為使用chat-GPT輔助教學相較於傳統老師授課，我更有信心能學好老師教授的知識與技能 *

單選。

	非常不同意
1	○
2	○
3	○
4	○
5	○
	非常同意

14。 我認為使用chat-GPT輔助教學相較於傳統老師授課，我更有自信能學好程式語法與資料分析 *

單選。

	非常不同意
1	⬭
2	⬭
3	⬭
4	⬭
5	⬭
	非常同意

三、滿意度

15。 我認為使用chat-GPT輔助教學相較於傳統老師授課更讓我滿意 *

單選。

	非常不同意
1	⬭
2	⬭
3	⬭
4	⬭
5	⬭
	非常同意

16。 我認為使用chat-GPT輔助教學提供的資訊符合個人所需且令人滿意 *

單選。

	非常不同意
1	⬭
2	⬭
3	⬭
4	⬭
5	⬭
	非常同意

其他

17。 您對 chat-GPT應用於程式語言學習是否有其他想法或建議?請暢所欲言 *

Google 並未認可或建立這項內容。

Google 表單
